# Exploring the Metabolic Impact of Traumatic Brain Injury in CCI Mouse Models: A Focus on Early and Prolonged Injury Responses

**DOI:** 10.3390/ijms27146144

**Published:** 2026-07-09

**Authors:** Mohammad Mehdi Banoei, Brittney N. V. Scott, Brent W. Winston

**Affiliations:** 1 Department of Critical Care Medicine, Health Research Innovation Center (HRIC), University of Calgary, Room 4C64, 3280 Hospital Drive N.W, Calgary, AB T2N 4Z6, Canada; mmbanoei@ucalgary.ca (M.M.B.);; 2 Department of Bio-Medical Engineering, University of Calgary, Calgary, AB T2L 0Y2, Canada; 3 Departments of Medicine, Biochemistry and Molecular Biology, University of Calgary, Calgary, AB T2N 2T9, Canada

**Keywords:** traumatic brain injury (TBI), plasma metabolomics, controlled cortical impact (CCI) mouse model, metabolic profiling, HILIC–MS, biomarker discovery

## Abstract

Traumatic brain injury (TBI) disrupts brain metabolism, which evolves over time and varies with the severity of the injury. Monitoring these metabolomic changes may reveal biomarkers indicating early damage, mechanisms of injury, and potentially help predict outcomes. This study used untargeted plasma metabolomics to investigate systemic time-dependent metabolic changes in mice exposed to controlled cortical impact (CCI) with or without replacement of a modified skull cap designed to reduce compensatory space for cerebral edema modelling a severe closed skull TBI, compared to sham controls. Male mice were subjected to CCI, CCI + CAP, or sham procedures comprised a scalp incision or a craniotomy. Plasma samples were collected at 4, 8, and 16 h, and 3 and 7 days after injury. Hydrophilic interaction liquid chromatography–mass spectrometry (HILIC-MS) was used to profile metabolites in all groups and time points, while ion-pair liquid chromatography–mass spectrometry (RPIPLC-MS) was used in CCI and sham mice at the early time points. The largest metabolic changes occurred at 8 h post-injury, distinguishing mice with CCI from sham controls. The early changes concerned metabolism of amino acids, energy, and nucleotide pathways, with metabolites such as succinate, phenylalanine, and cytidine showing significant changes. By 7 days, the metabolic patterns of the injured mice, especially CCI mice, had partially converged toward the sham state, although oxidative and mitochondrial disturbances persisted. The CCI + CAP mice had more pronounced and persistent metabolic disturbances compared to the CCI mice, which may reflect the effect of increased intracranial pressure post-injury. Plasma metabolomics can efficiently capture the evolving biochemical effects of TBI. The findings identified circulating metabolites that were associated with progression and severity of brain injury and provide a basis for future translational studies in human TBI.

## 1. Introduction

Traumatic brain injury (TBI) is the leading cause of disability and death worldwide, affecting men and women of all ages [1]. TBI is a highly heterogeneous disorder due to the inherent complexity of the brain and the diverse cellular and molecular mechanisms involved [2,3]. Primary mechanical injury can initiate a cascade of events, including metabolic dysfunction, oxidative stress, mitochondrial impairment, excitotoxicity, and neuroinflammation [4]. These processes can evolve dynamically over time and influence neurological recovery [4,5]. Metabolomics is a powerful tool to comprehensively study small-molecule metabolites and capture global biological changes [6].

The use of animal models can minimize the variability that is seen in human TBI and provide insight into specific metabolic responses during the initial and subsequent stages of TBI. A better understanding of these mechanisms may help to identify therapeutic modalities and guide the prognosis of patients with TBI. Given the relative inaccessibility of brain tissue for sampling in human neurological disorders such as TBI, metabolomics is more feasible using biofluids such as blood (serum or plasma) [7,8]. The controlled cortical injury (CCI) model is a validated and reliable model of TBI. CCI generates a consistent cortical injury through precise control of velocity, depth, and duration of tissue deformation in animals [9]. The cortical injury has three main effects: injury related to swelling of the brain, neuroinflammatory reactions and blood–brain barrier (BBB) damage. CCI provides replicable, precise control of impact parameters and severity, enabling researchers to study both focal injury and diffuse axonal injury (DAI) [10,11].

Controlled cortical impact is widely used in experimental TBI research because it produces a reproducible cortical injury through controlled biomechanical parameters, including impact velocity, penetration depth, dwell time, and impact location [9,10,11,12,13,14,15]. Recent studies and reviews further emphasize that CCI can reproduce several important pathological features of TBI, including cortical contusion, BBB disruption, edema, neuroinflammation, neurodegenerative changes, and behavioral or cognitive impairment [14,15,16]. These injury-related processes are closely linked to secondary biochemical cascades, including mitochondrial dysfunction, oxidative stress, excitotoxicity, amino acid imbalance, nucleotide turnover, and lipid remodeling. Therefore, metabolomics provides a valuable approach to capture the dynamic biochemical consequences of CCI injury and to identify circulating metabolic signatures associated with injury severity, progression, and recovery [7,8,17,18].

In this study, we used non-targeted liquid chromatography and mass spectrometry (LC-MS) metabolomics to investigate systemic metabolic changes in a severe CCI mouse model with or without a modified skull cap to simulate increased intracranial pressure post-TBI. The main aim of the study was to identify temporal metabolic signatures associated with injury and recovery, with relevance for biomarker discovery and translational TBI research.

## 2. Results

**Figure 1:**
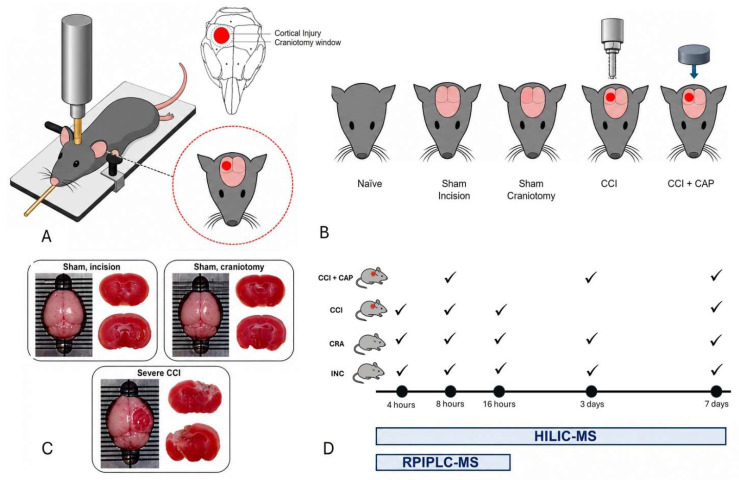
Experimental design and animal models of traumatic brain injury. (**A**) Schematic representation of the controlled cortical impact (CCI) procedure, showing the surgical setup and impact site on the exposed cortex. (**B**) Experimental groups included naïve controls, sham incision, sham craniotomy, CCI, and CCI combined with CAP replacement to mimic TBI with higher ICP (CCI-CAP). (**C**) Representative brain images from sham incision, sham craniotomy, and severe CCI mice illustrate the extent of the cortical damage. (**D**) Study design for plasma metabolomics analysis. Plasma samples were collected at 4, 8, 16 h, 3 days, and 7 days post-injury across the experimental groups. HILIC-MS analysis was performed for all groups and all time points, whereas RPIPLC-MS analysis was restricted to CCI and sham animals at 4 h, 8 h, and 16 h. Representative gross/microscopic brain images demonstrate intact brain tissue in sham incision and sham craniotomy animals and clear cortical damage in severe CCI animals, supporting the successful establishment of the injury model.

Successful establishment of the CCI model was supported by representative gross brain and coronal section images showing clear cortical tissue damage in severe CCI animals. In contrast, sham incision and sham craniotomy animals showed preserved brain morphology without comparable cortical disruption (Figure 1C). These observations, together with the use of standardized CCI impact parameters, support successful induction of cortical injury in the CCI model.

### 2.1. Distinct Systemic Metabolic Profiles Were Observed at 8 H Post-Brain Injury

Metabolomic analysis of the CCI mouse model showed that the most pronounced plasma metabolic changes occurred at 8 h post-injury, compared to 4 h, 16 h, and 7 days. At this time point, metabolite profiles identified by HILIC-MS were clearly distinct from those at 4 and 16 h (Figure S1). Consistent results were observed between RPIPLC-MS and HILIC-MS at 8 h, and both platforms showed significant differences between CCI and sham controls (Figures S2 and S4).

A total of 45 metabolites overlapped between the two analytical platforms, of which 32 showed consistent trends, and 23 were correlated by covariation analysis (Supplementary Figures S1 and S2). This overlap reflects cross-platform detection and does not imply that all metabolites were significantly altered at 8 h post-injury.

**Figure 2:**
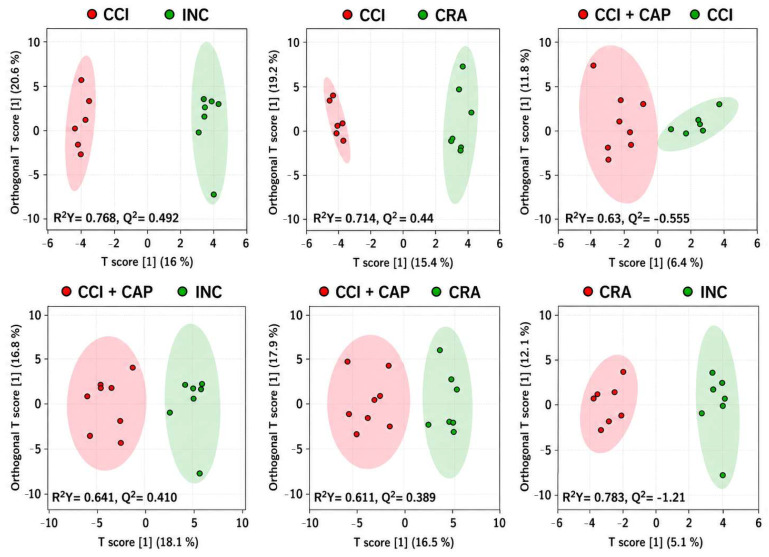
OPLS-DA score plots of plasma metabolites obtained using the HILIC-MS database in CCI, CCI-CAP, and sham control (CRA, INC) groups. The metabolic profiles of CCI and CCI + CAP mice showed predictive separation from sham controls (Q^2^ > 0.3), indicating distinct metabolic alterations. In contrast, the comparisons between CCI and CCI-CAP and CRA and INC were not predictive (Q^2^ = –0.555 and –1.21, respectively), despite the visual separations observed in the score plots.

At 8 h, both CCI and CCI-CAP models exhibited metabolic profiles significantly different from sham controls (INC and CRA) (Figure 2 and Figure S3), while no significant differences were observed between CCI and CCI + CAP. Similarly, CRA and INC showed comparable metabolic phenotypes (Figure 2 and Figure S3). PCA (Figure S3) demonstrated clear separation between injury models and sham controls, which was further supported by permutation testing (999 iterations) confirming statistical significance with less likelihood of overfitting of the data.

OPLS-DA analysis based on HILIC-MS data at 8 h further supported these findings, showing that CCI and CCI + CAP models were predictive and distinguishable from sham controls (Q2 > 0.3). In contrast, comparisons between CCI vs. CCI + CAP and CRA vs. INC were not predictive (Q2 = −0.555 and −1.2, respectively), despite apparent visual separation in the plots (Figure 2).

**Figure 3:**
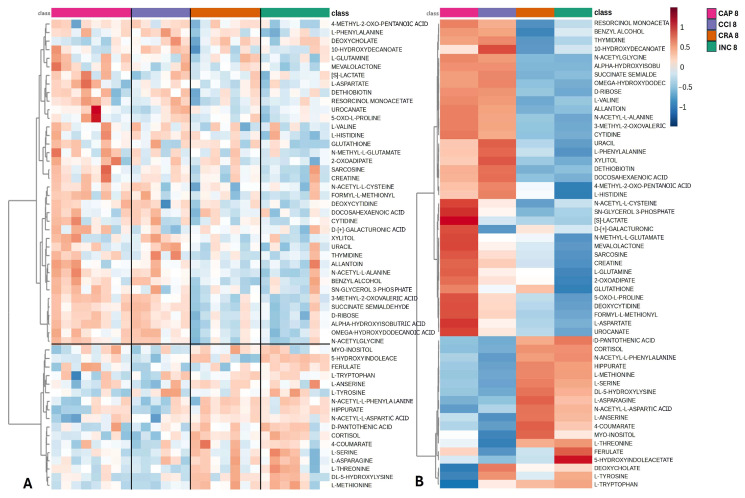
HILIC-MS-based heatmap analysis of plasma metabolomic profiles at 8 h post-injury. Panel (**A**) shows metabolite abundance patterns across individual animals in the CCI, CCI + CAP, CRA, and INC groups. Panel (**B**) shows the averaged metabolite profile for each group, summarizing group-level metabolic differences. The heatmaps highlight shared and distinct metabolic alterations between injury groups and sham controls and show that the sham INC and CRA groups have similar metabolic profiles, which differ from the injury models.

The *t*-test analysis (Tables S1–S4) revealed that injury models showed elevated levels of succinate semialdehyde, D-ribose, alpha-hydroxyisobutyric acid and 3-methyl-2-oxovaleric acid compared to controls, which indicate early mitochondrial stress and carbohydrate metabolism disturbances. The metabolites linked to amino acid metabolism showed decreased levels in both CCI and CCI + CAP groups when compared to sham controls with significant reductions in L-methionine, N-acetyl-L-aspartic acid and N-acetyl-L-phenylalanine (Tables S1–S4). The aromatic amino acids L-phenylalanine and L-histidine showed increased levels in CCI and CCI + CAP compared to sham controls, but L-tryptophan levels decreased only in CCI + CAP compared to sham controls. The metabolic changes in lipid and oxidative stress markers showed identical patterns between the two injury models because omega-hydroxydodecanoic acid levels increased while ferulate and cortisol concentrations decreased in both CCI and CCI + CAP groups relative to sham controls at 8h post-injury. The three metabolites N-acetylglycine, omega-hydroxydodecanoic acid and succinate semialdehyde showed increased levels in both injury models, which indicates they share a common immediate metabolic response to injury. Figure 3 shows the hierarchical clustering heatmap, a multivariate analysis between groups of mice at 8 h, which identifies the most significantly changed metabolites. Mevalolactone, sarcosine, creatine, L-glutamine, 2-oxoadipate, glutathione, deoxycytidine, L-aspartate, and urocanate increased in the CCI mice and correlated in CCI + CAP mice, according to the average concentration of metabolites compared to controls (Figure 3B). The CCI + CAP metabolic profile was associated with changes in more metabolites than the CCI group, according to the multivariate approach.

### 2.2. CCI+CAP Mice Have Prolonged Metabolic Changes Compared to CCI Mice at 7 Days Post-Injury

**Figure 4:**
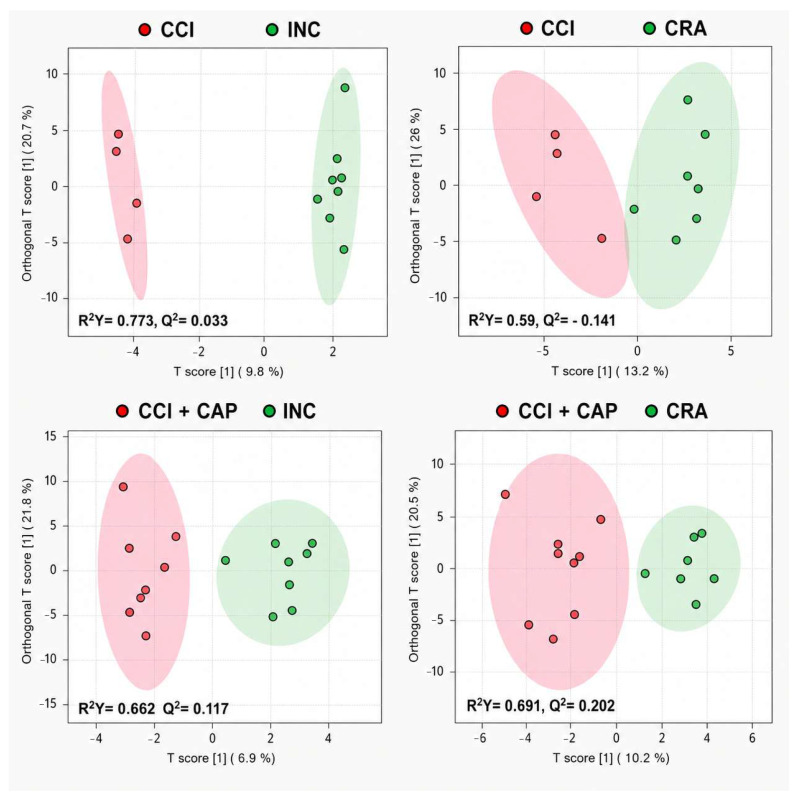
OPLS-DA plots of plasma metabolites at 7 days post-injury showed partial separation between the injury groups (CCI, CCI + CAP) and sham controls (CRA, INC) using HILIC data. While the overall predictive power was modest (Q^2^ < 0.3), CCI + CAP mice show clearer separation from shams than CCI mice, suggesting a more distinct metabolic phenotype at this later time point.

At 7 days post-injury, metabolomic analysis revealed that the CCI + CAP model exhibited broader and more persistent metabolic alterations than the CCI model, indicating greater or sustained injury. Compared with sham controls, CCI + CAP mice showed clear and statistically significant separation in metabolic profiles (*p* < 0.05; permutation test *p* < 0.01) (Figure 4). In contrast, the CCI group displayed only modest differences from sham animals, with not significant *p*-values of permutation tests, confirming a lack of predictive strength., although OPLS-DA plots visually differentiated the CCI and sham groups. The CCI + CAP mice showed a distinct and predictive metabolic signature relative to shams, suggesting that increased intracranial pressure amplified both the systemic and metabolic impact of the injury.

**Figure 5:**
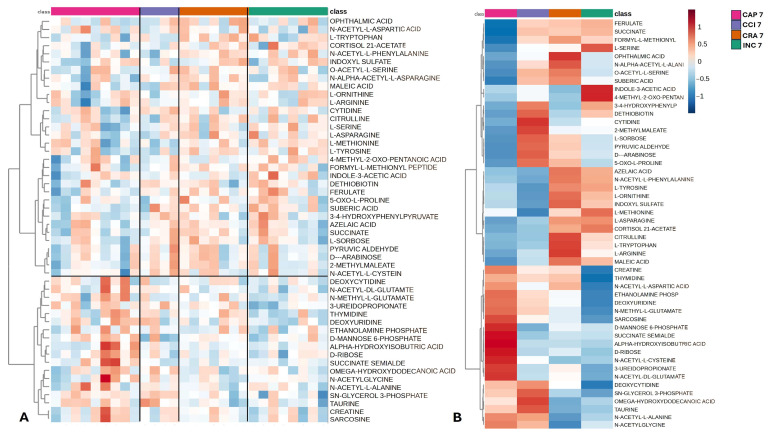
HILIC-MS-based heatmap analysis of plasma metabolomic profiles at 7 days post-injury. Panel (**A**) shows metabolite abundance patterns across individual animals in the CCI, CCI + CAP, CRA, and INC groups at 7 days post-injury. Panel (**B**) shows the averaged metabolite profile for each group, summarizing group-level metabolic differences. The heatmaps indicate that the CCI group shows partial convergence toward sham controls by 7 days, whereas the CCI + CAP group retains a more distinct metabolic profile, suggesting persistent metabolic alterations after injury.

**Figure 6:**
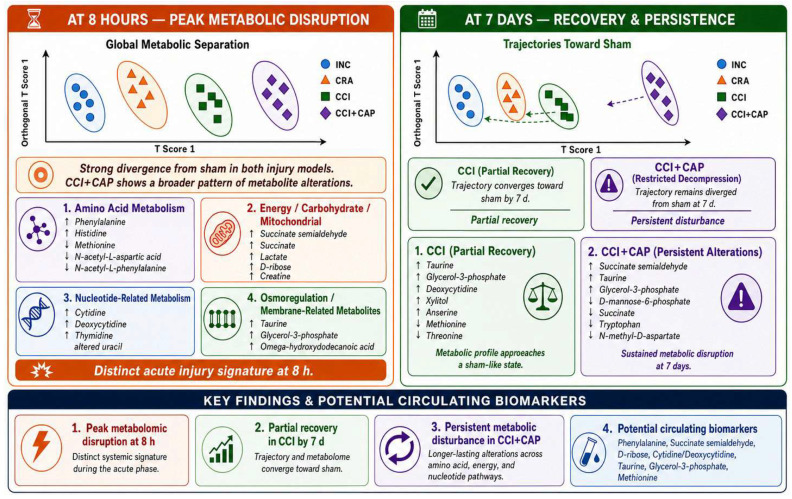
Schematic summary of time-resolved plasma metabolomic changes after CCI and CCI + CAP injury in mice. The figure highlights peak metabolic disruption at 8 h post-injury, partial recovery in CCI by 7 days, and persistent metabolic alterations in CCI + CAP, with candidate-circulating metabolites linked to injury severity and progression. ↑ indicate increased metabolite levels, ↓ indicate decreased metabolite levels. The ← indicates partial convergence of the metabolomic profile toward sham controls by 7 days. This convergence is more evident in the CCI group, whereas the CCI + CAP group shows a weaker shift toward sham and remains more metabolically distinct, consistent with persistent metabolic disturbance.

By 7 days, results confirmed that overall metabolic disturbances had significantly decreased compared with the acute 8 h phase, yet several metabolites remained altered (Tables S5–S8). At 7 days, in CCI mice, levels of xylitol, glycerol-3-phosphate, deoxycytidine, taurine, and anserine were elevated, while L-methionine and L-threonine were reduced, reflecting ongoing changes in amino acid and nucleotide metabolism. At 7 days, the CCI + CAP group showed increased succinate semialdehyde, D-mannose-6-phosphate, taurine, and glycerol-3-phosphate, alongside decreased succinate, L-tryptophan, and N-methyl-D-aspartate, suggesting partial recovery of energy pathways but continued dysregulation of excitatory amino acids. Both injury groups showed reduced N-acetyl-L-phenylalanine, L-threonine, L-tyrosine, and ascorbate compared with CRA shams, along with elevated taurine and glycerol-3-phosphate. Heatmap analyses (Figure 5A,B) further supported these findings, showing that CCI+CAP mice developed a distinct metabolic pattern, while the CCI and sham groups displayed relatively similar profiles by 7 days. Schematic summary of time-resolved plasma metabolomic changes after CCI and CCI + CAP injury in mice (Figure 6). The figure highlights peak metabolic disruption at 8 h post-injury, partial recovery in CCI by 7 days, and persistent metabolic alterations in CCI + CAP, with candidate-circulating metabolites linked to injury severity and progression.

**Figure 7:**
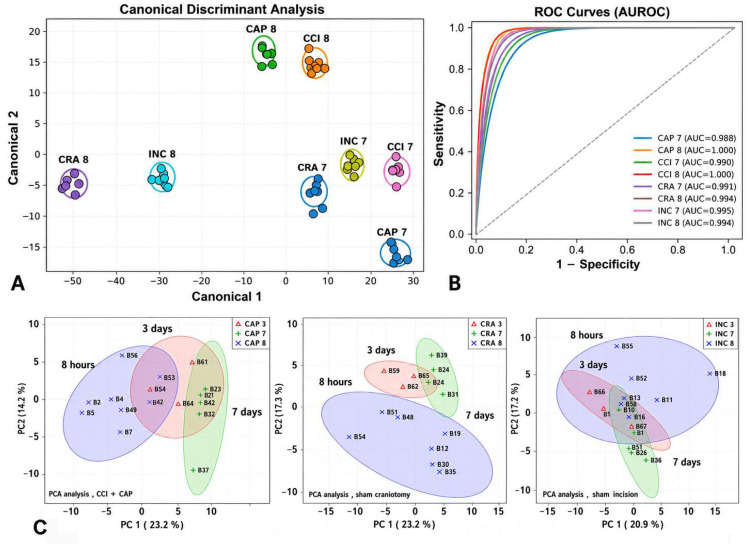
(**A**) HILIC data-based linear discrimination analysis showed that CCI 8 and CCI + CAP 8 were very different from sham craniotomy (CRA 8) and incision (INC 8) mouse metabolomics at 8 h post-injury. The metabolomics of CCI mice (CCI 7) were similar to those of sham controls (CRA 7 and INC 7) at 7 days post-injury, while the metabolites of CCI + CAP mice (CAP 7) were very different from those of sham controls (CRA 7 and INC 7) and CCI mice (CCI 7) at 7 days post-injury. (**B**) The AUROC was very high for each group at two time points (8 h and 7 days post-injury). The number following the abbreviation indicates the time (8 h and 7 d) after the brain injury. (**C**) PCA showed a change in the metabolic profile over time for CCI + CAP. There were fewer changes in metabolites in the sham craniotomy group than in the CCI + CAP group, but more than in the sham incision group.

Moreover, linear discriminant analysis (LDA) applied to HILIC metabolite profiles at 8 h post-injury achieved strong discrimination between CCI, CCI + CAP, and sham controls, reflecting pronounced injury-specific metabolic differences. The LDA showed that the CCI + CAP group maintained a greater distance from CCI and sham controls at 7 days post-injury, indicating that the CCI + CAP mice had more significant and prolonged metabolic changes due to the brain injury, while CCI mice had smaller distances from sham controls at 7 days, indicating a partial metabolic recovery. These findings highlight a pronounced metabolic response to brain injury compared to controls and highlight potential biomarkers for the monitoring of the progression of injury and recovery (Figure 7A).

### 2.3. Time Drift of Metabolite Alterations

In the CCI + CAP brain injury model, the PCA revealed a clear time-dependent metabolic drift with a gradual shift in metabolite profile from 8 h to 3 days and 7 days post-injury, indicating significant and dynamic changes based on the HILIC data (Figure 7B). On the other hand, sham craniotomy showed a moderate metabolic change over time, indicating a minor metabolic impact associated with the surgery, while sham incision showed a minimal change, suggesting a minor metabolic effect. The clear separation of CCI + CAP from the sham groups highlights the injury-specific metabolic alterations, while the slight separation of the sham craniotomy group suggests a modest metabolic alteration.

These findings highlighted a pronounced metabolic response to brain injury compared to sham surgery, highlighting potential biomarkers for the monitoring of the progression of injury and recovery in mice with TBI.

### 2.4. Global Metabolite Changes in Injury Models Compared to Sham Controls

Compared to sham controls, plasma metabolomics at 8 h following CCI and CCI + CAP injury revealed extensive disruptions of amino acid, energy, nucleotide, and lipid metabolisms. Several amino acids are involved in the metabolism of proteins, including phenylalanine, serine, aspartate, histidine, valine, and methionine. Energy-related metabolites, such as creatine, succinate, lactate, and mevalonolactone, increased, suggesting increased energy demand and mitochondrial stress. In accordance with DNA turnover and cellular stress, the nucleotide intermediates cytidine, deoxycytidine, thymidine, and uracil were also altered. Alpha-hydroxyisobutyrate, glutathione, and docosahexaenoic acid all indicated oxidative imbalance and lipid remodeling.

After 7 days, the levels of tryptophan, serine, arginine, asparagine, and methionine decreased in both injury models compared with controls. Increases in succinate, citrate, and suberic acid levels indicate changes in mitochondrial functions and energy metabolism. Nucleoside-related compounds such as cytidine, thymidine, deoxycytidine, and inosine derivatives indicate ongoing DNA stress response. Changes in metabolites related to lipids, such as alpha-tocopherol, indoxyl sulfate, and omega-hydroxydodecanoic acid, may reflect responses to oxidative stress and cell membrane alteration. Alpha-tocopherol, serine, succinate, and tryptophan were the main metabolites in separating the sham and injured animals at 7 days.

We noted similar findings when comparing two statistical approaches, univariate analysis and multivariate heatmap analysis. Both approaches to brain injury showed that methionine, succinate semialdehyde, D-ribose, alpha-hydroxyisobutyrate, N-acetylglycine, and omega-hydroxydodecanoic acid consistently increased in the injury groups, while N-acetyl-aspartic acid, N-acetyl-phenylalanine, and cortisol decreased. These repeating metabolite patterns, demonstrated using different analytic techniques, helped confirm the validity of the metabolic signatures associated with brain injury. While univariate analysis revealed small but significant changes in individual metabolites, the heatmap revealed a more extensive clustering pattern.

## 3. Discussion

We show here that induced brain injury can dramatically alter the plasma metabolome in a mouse model of severe TBI compared to sham controls. Importantly, the largest metabolic divergence occurred at 8 h post-injury, indicating an acute biochemical crisis that was partially resolved by 7 days. This emphasizes the short timeframe during which primary and early secondary metabolic changes dominate, suggesting an important time window for biomarker discovery and early therapeutic intervention. The 8 h time point revealed extensive alterations in energy and amino-acid metabolism, including elevations in succinate, lactate, phenylalanine, cytidine, and glutamate, consistent with mitochondrial dysfunction, glycolytic acceleration, and excitatory stress. These early disturbances likely represent the phase of rapid oxidative disruption and initiation of secondary biochemical cascades. By 7 days, many abnormalities are attenuated in the CCI group, with partial normalization of TCA-cycle intermediates and amino acids. However, residual increases in metabolites such as taurine, glycerol-3-phosphate, and succinate semialdehyde indicate incomplete recovery and persistent oxidative stress. Together, these temporal shifts outline a clear progression from acute metabolic crisis toward a slower recovery stage. Consistent with our previously described human severe TBI metabolomics studies, several metabolites displayed concordant alterations between human severe TBI patients and mice with CCI, including increased phenylalanine, lactate, asparagine, and alanine, and reduced methionine and threonine [18].

From a translational perspective, the early plasma metabolic changes observed at 8 h post-injury may have potential value for the development of diagnostic and prognostic biomarker panels in TBI. These metabolites may complement neurological assessment, neuroimaging, and established or emerging protein biomarkers by reflecting downstream biochemical consequences of brain injury, including mitochondrial stress, oxidative injury, amino acid imbalance, nucleotide turnover, lipid remodeling, and neuroinflammation [7,8,17,18,19,20,21,22]. In particular, metabolites that changed consistently across injury models and analytical approaches may be useful candidates for future validation as early indicators of injury-associated metabolic disruption, while persistent changes at 7 days may be relevant for monitoring prolonged injury responses or incomplete metabolic recovery. However, translation to clinical practice requires caution because plasma metabolites may be influenced by systemic inflammation, anesthesia, diet, renal or hepatic metabolism, sample handling, and non-neurological injuries. Therefore, the candidate metabolites identified in this study should not be interpreted as ready-to-use clinical biomarkers, but rather as discovery-stage signatures requiring targeted quantitative validation. A notable finding of this study is the time-dependent nature of the metabolic response, with the most pronounced systemic metabolic alterations observed at 8 h post-injury. This early time window may be particularly important translationally, as most human severe TBI metabolomics studies have not captured samples within the first 8 h after injury, leaving this acute metabolic phase relatively underexplored.

Future translational studies should validate selected metabolites using targeted LC-MS/MS assays in independent animal cohorts and human TBI samples, examine their association with injury severity and functional outcomes, and evaluate whether metabolite panels add diagnostic or prognostic value beyond current clinical models, imaging findings, and protein biomarkers such as GFAP and UCH-L1 [8,17,18,23]. In addition, clinically feasible implementation will require prioritizing metabolites that are stable in plasma, reproducibly measurable, biologically interpretable, and suitable for conversion into standardized laboratory assays or point-of-care platforms. Such validation would help determine whether metabolite-based signatures can be used not only for biomarker discovery but also for patient stratification, monitoring of injury progression, and future therapeutic-response assessment.

The placement of a modified skull cap after craniotomy profoundly influenced the metabolic trajectory. The CCI + CAP model showed more pronounced and sustained metabolic deviations from sham and CCI mice at both 8 h and 7 days, suggesting a more severe and prolonged injury state. We have demonstrated that CCI + CAP mice have significantly worse neurological deficits compared to CCI alone when scored on aspects such as gait, posture, and grasp (unpublished data). Although intracranial pressure (ICP) was not directly measured, the cap likely restricted compensatory brain expansion, increased intracranial pressure, and impaired perfusion. Clinically, this is relevant for patients with raised ICP after severe TBI, where early decompressive strategies may mitigate similar metabolic crises. Therefore, the CAP model should be interpreted as a modified injury model that restricts the craniotomy-related decompressive space, rather than as a directly measured ICP model.

In our study, brain injury showed increased systemic levels of n-acetylglycine, n-acetyl-alanine, allantoin, succinate semialdehyde, phenylalanine, ɑ-hydroxybutyric acid, 2-oxoadipate, deoxycytidine, sn-glycerol-3-phosphate, and creatine, all of which indicate increased oxidative stress. Invasive damage to brain tissue results in direct DNA damage and destruction of tissue [24,25], consistent with the changes observed in DNA-related metabolites such as deoxycytidine and thymidine. An increase in sn-glycerol-3-phosphate levels is directly related to glycerophospholipid metabolism and likely arises from the breakdown of phosphatidylcholine (PC) following injury. This mirrors findings in our previous study including human severe TBI patients with poor outcomes [8].

Brain injury models showed increased energy-related metabolites and pentose phosphate pathway intermediates, including ribose and other sugars. Levels of succinate semialdehyde, uracil, and thymidine increased at different times in the CCI and CCI + CAP models, indicating metabolic disturbances of GABAergic and pyrimidine pathways. These patterns illustrate how multiple biochemical systems, energy, amino acids, and nucleotides remain interlinked during the injury and recovery process. Biochemical pathways indicate that severe TBI injuries induce specific metabolic changes that are consistent between humans and mice, regardless of patient factors or the site or spread of damage. Our findings agree with other studies showing increased succinate semialdehyde levels in CCI models, suggesting that the pathophysiology of TBI is closely tied to mitochondrial dysfunction and oxidative stress [26,27]. Injury models also showed increased D-ribose, consistent with studies reporting pentose phosphate pathway activation as a protective mechanism for DNA repair. Similar trends were observed in our prior human severe TBI analyses [18] where we showed increases in alpha-hydroxyisobutyric acid from BCAA breakdown that we also see due to the brain injury in CCI and CCI + CAP models, suggesting that amino acid metabolism remains impaired as mitochondrial damage shifts energy reliance to alternative substrates [19,22]. The current study further revealed significant alterations in methylation pathways and antioxidant defense systems. L-methionine, an essential amino acid required for methylation and glutathione synthesis, was reduced in all models. This depletion is consistent with oxidative stress and disruption of one-carbon metabolic pathways. Increased N-acetylated amino acids and glutathione reflect active detoxification processes that protect neurons against oxidative injury. Together, these metabolite shifts underscore the persistent biochemical stress experienced by injured tissue even after initial stabilization. It has been shown that N-acetylation and glutathione conjugation protect neurons against oxidative damage occurring after injury. Increased xylitol, sn-glycerol 3-phosphate, and taurine levels at 7 days post-injury are consistent with prolonged osmotic stress, lipid remodeling, and neuroinflammatory patterns observed in longitudinal metabolomics studies of TBI [22,27].

In brain-injured animals, decreases in glucose-1-phosphate and changes in lactate and succinate reflect disturbances in energy production [27,28]. The neurotransmitter system is impaired, with increased glutamate and reduced GABA, suggesting excitotoxic pressure on vulnerable neurons [20,28]. Increased ADP, AMP, NAD+, and IMP point to purine pathway disturbances and depletion of nucleotide pools [20]. Alterations in taurine, creatinine, and branched-chain amino acids indicate stress on osmotic regulation and amino acid metabolism [27,28]. Reduced glutathione and elevated ROS signal oxidative stress and associated inflammatory responses [29,30]. Additional brain-region variability has been reported, with the hippocampus showing oxidative stress and ion pump disruption [20,21] and the thalamus displaying choline and glycerophospholipid changes [28,31]. Time-dependent metabolic differences are also well recognized, with early energy disturbances followed by longer-term shifts in amino acid and lipid metabolism [26,32]. Acute-phase alterations in linoleic acid, proline, phosphoric acid, and β-hydroxybutyric acid have been seen in plasma and serum metabolomics post-injury [19]. While less acute changes in methionine sulfoxide, kynurenine, and glycocholic acid highlight the evolution of injury timing [21,33].

This study should be interpreted as a discovery-oriented plasma metabolomics analysis rather than a definitive mechanistic validation study. Although the observed metabolic alterations are biologically consistent with known secondary injury processes after TBI, including mitochondrial dysfunction, oxidative stress, excitotoxicity, amino acid dysregulation, nucleotide turnover, lipid remodeling, and neuroinflammation [4,7,8,17,18,19,20,21,22,27,28,34], these pathway-level interpretations remain inferential. The present study did not directly correlate plasma metabolite levels with quantitative structural or functional endpoints, such as lesion volume, histology, neurobehavioral scores, intracranial pressure, or neuroimaging measurements. In addition, although representative brain images and standardized CCI parameters support successful induction of injury, formal grading of injury severity ideally requires integration of impact parameters with structural and behavioral outcomes [9,10,11,12,13,14]. Another limitation is that the candidate metabolites were identified through untargeted LC-MS profiling and were not independently verified using targeted quantitative assays or authentic standard mimics according to formal metabolomics identification and quantitative reporting standards [35]. Therefore, the metabolites reported here should be considered candidate injury-associated metabolic signatures rather than fully validated mechanistic biomarkers. Future studies should combine untargeted and targeted LC-MS/MS metabolomics with histological assessment, lesion-volume quantification, neurological scoring, ICP monitoring, imaging, and comparison with established TBI protein biomarkers to determine how specific circulating metabolites relate to structural injury, functional impairment, and clinical outcome.

There are a number of limitations of this study that should be acknowledged. The CCI model cannot fully reproduce the complexity and heterogeneity of human TBI, especially diffuse axonal injury and behavioral outcomes. Translating metabolite findings from mice to humans remains challenging due to interspecies metabolic differences. Plasma metabolomics may not fully reflect local brain changes due to systemic compensation. The potential influence of anesthesia on TBI outcomes should be considered, as ketamine has been reported to have neuroprotective effects by modulating excitotoxicity and cerebral metabolism [36,37]. However, uniform anesthetic exposure across all animal groups controls for this variable. The number of animals per time point may limit the detection of subtle changes. Moreover, only male mice were used, which may have overlooked sex-related differences. Despite these limitations, the study provides important evidence of early significant metabolic changes 8 h after brain injury in CCI mice. These results provide a strong basis for future validation in larger and more diverse cohorts.

## 4. Methods and Materials

### 4.1. Animal Model and Experimental Design

All procedures were approved by the Animal Care Committee at the University of Calgary (AC16-0144). Ninety male C57BL/6 mice (8 to 10 weeks of age) obtained from Jackson Laboratory were housed in standard conditions with free access to food and water. Four experimental groups were investigated: CCI, CCI with a modified skull cap to limit brain expansion likely resulting in increased intracranial pressure (CCI + CAP), sham craniotomy (CRA), and sham incision (INC). Plasma was collected at 4, 8, and 16 h, as well as 3 and 7 days post-injury, respectively. Each time point included 4 to 9 mice per group. The initial analyses focused on the CCI, CRA, and INC models at 4 to 16 h, and the CCI+CAP group was included at 8 h, 3 days, and 7 days.

The experimental groups were designed to distinguish brain injury-related metabolic changes from changes caused by anesthesia, incision, craniotomy, and surgical manipulation. The CCI group represented the standard controlled cortical impact model, which produces reproducible focal cortical injury using controlled biomechanical parameters [9,10,11,12,13]. The CCI+CAP group received the same cortical impact, followed by the replacement of a cap over the craniotomy site to reduce the artificial decompressive space created by skull opening. This modification was intended to better approximate aspects of severe TBI in which restricted brain expansion and increased intracranial pressure may contribute to secondary injury. The sham craniotomy group controlled for the effects of craniotomy and surgical manipulation without cortical impact, whereas the sham incision group controlled for anesthesia, scalp incision, handling, and postoperative recovery without craniotomy or direct brain injury [29,30].

### 4.2. Controlled Cortical Impact Model

The CCI model is an established model of traumatic brain injury. To induce a reproducible injury, a craniotomy is performed to allow direct impact onto the cortical surface. A limitation of this method is that it may not fully recapitulate a severe closed-skull TBI, in which increased intracranial pressure can play a central role in secondary injury. Our research group developed a modified CCI model in which a “cap” or plug is placed immediately after cortical impact to remove the compensatory space created by craniotomy and better model closed-head injury.

Mice were anesthetized by intraperitoneal injection of ketamine (Bayer Animal Health, Leverkusen, Germany) (200 mg/kg) and xylazine (Bimeda-MTC Animal Health Inc., Cambridge, ON, Canada) (10 mg/kg). After shaving the head and fixing the head in a stereotaxic frame, the skin (scalp) was incised, and a 5 mm right lateral craniotomy was performed using a motorized drill (Fine Science Tools Inc., Foster City, CA, USA). Controlled cortical impact was induced using an electromagnetically controlled Impact One Stereotaxic Impactor (ImpactOneTM Stereotaxic Impactor, Leica Microsystems, St. Louis, MO, USA) with a 3 mm-diameter tip, 4.0 m/s impact velocity, 2.5 mm impact depth, and 0.1 s dwell time. After injury, a sterile plastic disc (for the CCI group) or cap (for the CCI + CAP group) was fastened to the skull over the impact site with Vetbond liquid tissue adhesive (3M Company, Saint Paul, MN, USA) and the skin sutured. Successful establishment of the CCI model was supported by the use of standardized stereotaxic injury parameters and representative gross brain images showing clear cortical damage in injured animals compared with intact brain tissue in sham incision and sham craniotomy controls (Figure 1C). The impactor system allowed controlled delivery of injury through defined impact velocity, depth, dwell time, and tip diameter, which are central advantages of the CCI model for generating reproducible experimental TBI [9,10,11,12,13]. The CCI mouse model was established based on previously described murine traumatic brain injury procedures reported by Nguyen et al. [38]. In addition, the absence of comparable tissue disruption in the sham incision and sham craniotomy groups supports that the observed injury pattern was caused by cortical impact rather than anesthesia, incision, or craniotomy alone.

Sham craniotomy and incision controls underwent equivalent procedures without an impact causing brain injury (Figure 1). After surgery, saline was administered subcutaneously, and the mice were monitored during the post-operative recovery [12,13]. Blood was taken from each mouse at a defined time upon sacrifice by cardiac puncture. Plasma was prepared in heparin tubes by centrifugation. All samples were treated identically and frozen to −80 °C for storage and thawed at 4 °C for metabolite determination.

### 4.3. Sample Preparation and Metabolite Extraction

To reduce pre-analytical and technical variation, all plasma samples were processed using a standardized workflow. Once blood samples were taken, they were placed on ice, centrifuged at 4 °C to isolate plasma, then aliquoted and immediately stored at −80 °C for storage. For metabolomics analysis, the sample aliquots were thawed once to minimize repeated freeze–thaw cycles [31]. Samples were randomly ordered before LC-MS acquisition to reduce systematic run-order bias. For metabolite extraction, 50 µL of plasma was mixed with 150 µL of pre-cooled 50% methanol, vortexed, and centrifuged at 13,000× *g* for 10 min at 4 °C. The supernatant was diluted 1:20 using 50% methanol and transferred to 96-well plates for LC-MS analysis [39]. Pooled quality control samples, prepared from equal volumes of multiple study samples, were included during MS analysis to monitor instrumental and analytical variation. Metabolite peaks were further evaluated using ion intensity and comparison with pre- and post-blank samples during data processing.

### 4.4. Metabolomics Analysis

Metabolomic profiling was performed using a Q Exactive HF Hybrid Quadrupole-Orbitrap mass spectrometer (Thermo Fisher Scientific, Mississauga, ON, Canada). HILIC-MS and RPIPLC-MS were used as complementary LC-MS approaches to improve metabolite coverage rather than as duplicate analytical methods. Because metabolites differ substantially in polarity, charge, hydrophobicity, and chromatographic retention behavior, no single LC-MS platform can capture the full biochemical diversity of the plasma metabolome. HILIC-MS was selected as the primary platform across all groups and time points because it is particularly useful for retaining and detecting hydrophilic and polar metabolites, including amino acids, nucleosides, organic acids, sugars, and other small polar compounds [39,40,41]. In contrast, RPIPLC-MS was used as a complementary acute-phase platform because ion-pairing reverse-phase chromatography improves retention and separation of highly polar or charged metabolites that are often poorly retained by conventional reverse-phase LC, including organic acids, nucleotide-related compounds, sugar phosphates, and central-carbon metabolites [42,43,44]. Therefore, HILIC-MS provided broad longitudinal profiling across the experimental design, whereas RPIPLC-MS added complementary biochemical coverage during the early post-injury phase. RPIPLC-MS was restricted to the 4, 8, and 16 h samples because its purpose was to support and expand the acute-phase findings, particularly around the 8 h window where the strongest metabolic divergence was observed. The delayed 3- and 7-day analyses were performed using HILIC-MS, which was applied consistently across all groups and time points and therefore provided the primary framework for temporal comparisons.

Data were acquired in positive and negative ionization modes and processed using El-MAVEN, version 0.12.0 [45] and MetaboAnalyst 6.0 [46]. Statistical analyses included Student’s *t*-tests, analysis of variance (ANOVA), principal component analysis (PCA), orthogonal partial least squares discriminant analysis (OPLS-DA), and Receiver Operating Characteristic (ROC) curve evaluation. Corrected *p*-values < 0.05 were considered significant. Linear Discriminant Analysis (LDA) is a supervised machine learning method used for classification and dimensionality reduction. It separates multiple groups by maximizing the distance between them, where larger distances in the plot indicate stronger discrimination between groups [47]. Detailed descriptions of experimental procedures, LC-MS parameters, and data processing workflows are provided in the Supplementary Materials.

## Data Availability

The raw data supporting the conclusions of this article will be made available by the authors on request.

## Supplementary Materials

The following supporting information can be downloaded at: https://www.mdpi.com/article/10.3390/ijms27146144/s1. References [48,49] are cited in the Supplementary Materials.
