# Supplementary material for: Exploring the Metabolic Impact of Traumatic Brain Injury in CCI Mouse Models: A Focus on Early and Prolonged Injury Responses
Source: Int J Mol Sci. 2026 Jul 9;27(14):6144. doi: 10.3390/ijms27146144 (PMC13410146; doi:10.3390/ijms27146144)
Supplement: Supplementary file 1 [file ijms-27-06144-s001.zip › ijms-4383255-supplementary.pdf]

Exploring the Metabolic Impact of Traumatic Brain Injury in CCI Mouse Models: A Focus on Early and Prolonged Injury Responses

Mohammad Mehdi Banoei[1,2], Brittney N.V. Scott[1], Brent W. Winston[1,3] on behalf of the CTRC (Canadian TBI Research Consortium) and the CCCTBG (Canadian Critical Care Translational Biology Group)

1 Department of Critical Care Medicine, University of Calgary, Calgary, AB, Canada.

2 Department of Bio-Medical Engineering, University of Calgary, Calgary, AB, Canada.

3 Departments of Medicine and Biochemistry and Molecular Biology, University of Calgary, Calgary, AB, Canada

Address correspondence to: Brent W. Winston,

Full address: University of Calgary, Health Research Innovation Center (HRIC), Room 4C64, 3280 Hospital Drive N.W., Calgary, Alberta, Canada, T2N 4Z6. Tel: (403) 220-4341, Fax: (403) 283-1267

Email: bwinston@ucalgary.ca

Sample Preparation

For the extraction of metabolites, 150 µL of 50% methanol was added to 50 µL of plasma samples from the animal. The methanol solution was pre-cooled to enhance the extraction efficiency, and the samples were centrifuged at 13,000 × g for 10 min at 4°C. A dilution of 1:20 was made from all samples by adding a 50% methanol solution. The samples were transferred to a 96-well plate for LC-MS analysis. (*3*).

Hydrophilic Interaction Liquid Chromatography (HILIC) Mass Spectrometry and metabolite profiling

The mouse TBI model samples were analyzed by UHPLC-MS (Q Exactive HF Hybrid Quadrupole-Orbitrap Mass Spectrometer, Thermo-Fisher, Canada). Ultra-high performance liquid chromatography was carried out using a 2.1 mm × 100 mm long Syncronis HILIC (Thermo-Fisher, Canada) LC column. The column has been packed with Hypercarb particles of 3 µm pore size. The mobile phase of the chromatography was carried out by gradient of acetonitrile elution with 0.1% formic acid for 20 min as follows: 95% for 2 min, 85%–95% for 5 min, 5%–80% for 3 min, 5% for 5 min, 5%–95% for 2 min, and then held at 95% for the remaining 3 min. The solvent A was 20 mM ammonium formate at a pH of 3.0 in MS-grade H_2O. The mass interference for HILIC-MS was performed by means of a Quadrupole-Orbitrap Mass Spectrometer under the following conditions: HESI-II temperature at 325°C, auxiliary gas flow 10 units, sheath gas flow 25, spray voltage ± 2.50 kV, capillary temperature 275°C, S-lens RF level 60%, and auxiliary gas heater temperature at 275°C for negative ion mode. Mass spectra were acquired using a 20-minute run time, full MS scan type with a resolution of 240,000, AGC target 3e6, maximum IT 200 ms, and

scan range 7-100 m/z (*3*). Maven software was used to process the HILIC-MS spectral data for metabolite identification and quantification. (*4, 5*). Briefly, metabolites or feature peaks were selected based on their ion intensity and ion signals in the pre- and post-blank samples run during the analysis.

RPIP-LC/MS Analysis

The Thermo Hypersil GOLD aQ C18 column (2.1 mm × 100 mm, 1.9 µm particle size) was used with a Thermo Scientific Vanquish UHPLC system for the separation of metabolites. The mobile phase system used tributylamine (TBA) as an ion-pairing agent in accordance with the method described by Rabinowitz et al. (2010). The mobile phase mixture consisted of water with 10 mM TBA and 15 mM acetic acid as its components. The mobile phase B consisted of methanol containing the same concentrations of TBA and acetic acid. The gradient started at 0–2 minutes with 0% B before linearly increasing to 95% B for 2–15 minutes and then maintaining 95% B for 15–17 minutes before reverting to 0% B for 3 minutes of re-equilibration. The system was operated at a 0.25 ml/min flow rate, and the column temperature was maintained at 40 °C. This method allows for the retention of diverse metabolites, including organic acids, nucleotides, sugar phosphates and other polar compounds.

Mass Spectrometry

Detection was performed using a Q Exactive HF Hybrid Quadrupole-Orbitrap Mass Spectrometer (Thermo-Fisher, Canada) equipped with a HESI-II ion source. The MS acquisition was performed in full-scan mode (m/z 70–1000) with a resolution of 120,000, with an AGC target of 3×10^6 and a maximum injection time of 200 ms. The ion source parameters were set up as follows: spray

voltage ±3.0 kV, sheath gas 40, auxiliary gas 10, capillary temperature 275 °C, S-lens RF level 60%, and auxiliary gas heater temperature 300 °C. Data were acquired in positive and negative ionic modes using separate injections to maximize coverage of the metabolites.

Data Analysis

El-MAVEN V.12 software by Elucidata Inc. (San Francisco, CA, USA) was used for the identification and quantification of metabolites by measuring the ion intensity of the compounds (*6*). The selection of the ion peaks for the analysis of metabolites was done by M/Z ratio and RT, and ion intensity was also compared with pre- and post-blank samples.

Principal component analysis (PCA) served as an unsupervised method for detecting outliers and provided a data overview. The orthogonal partial least discriminant analysis (OPLS-DA) was used to identify the different metabolites that differentiated the two groups and to evaluate the predictive power of the model based on Q2 values. Linear Discriminant Analysis (LDA) is a machine learning algorithm that acts as a supervised classification method that reduces dimensionality to handle datasets that contain multiple groups. The LDA can directly display the distance between groups by using a plot in which a large distance indicates a significant difference between the groups, which is a reflection of the discriminative power of the LDA. Non-parametric analysis of variance (ANOVA) was performed to detect differences between the samples of the different groups based on the raw ion intensities, and adjustment correction was applied to adjust for false discovery rate (FDR). The Student t-test was used to establish the difference between the two sample groups. All statistical tests were two-sided, and corrected p-values < 0.05 were considered statistically significant. The metabolites' diagnostic values were assessed by constructing receiver operating characteristic (ROC) curves and computing the area under the curve (AUC) and sensitivity at the

predefined specificities. Correlations between the variables of interest were tested by Spearman correlation and, if necessary, corrected for multiple comparisons using the Holm correction. A power analysis was performed to determine the minimum sample size necessary to detect a statistically significant difference between the two populations based on a level of confidence. For the comprehensive analysis of metabolomics data, MetaboAnalyst 6.0 (*7*), GraphPad Prism 9.5.1, and SIMCA P v 14.0 were used.

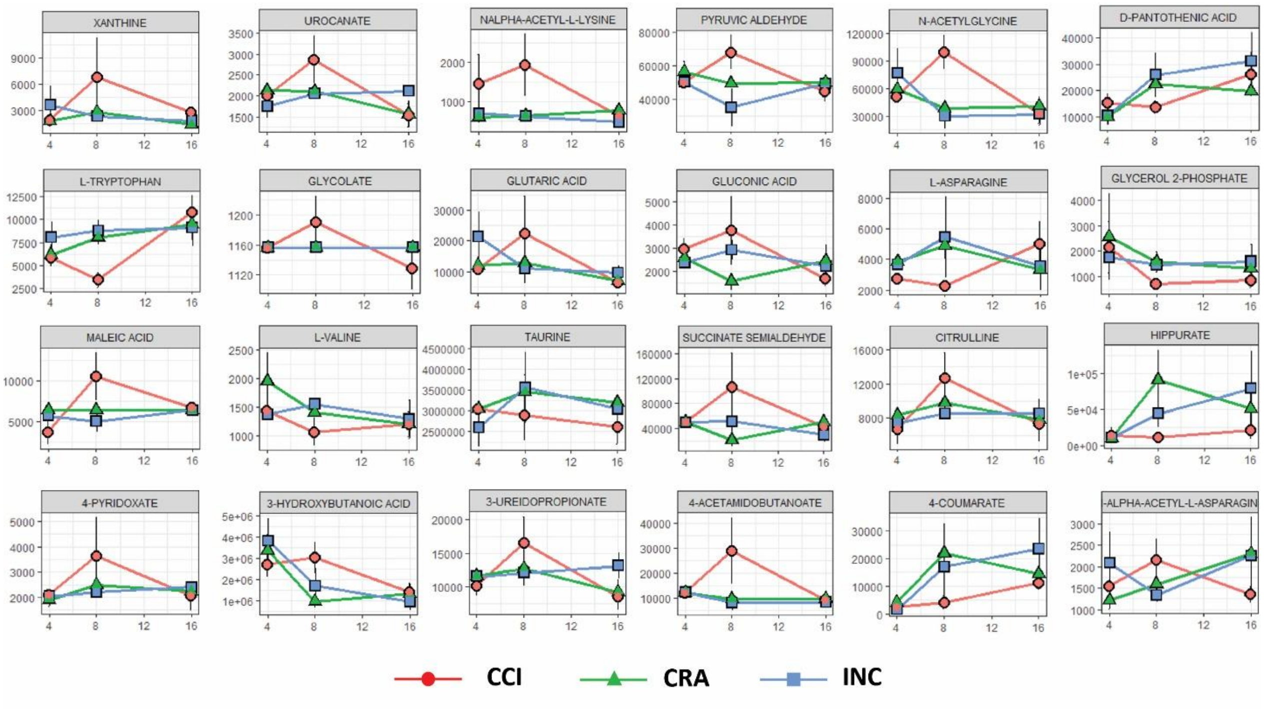

Figure S1. Plasma metabolite (HILIC-MS) profiles in the CCI mouse model of sTBI compared with sham controls (CRA, INC) across time points (4-16 h post-injury). The largest differences were detected at 8 h post-injury, where CCI mice showed clear divergence from controls, while 4 h and 16 h displayed smaller changes.

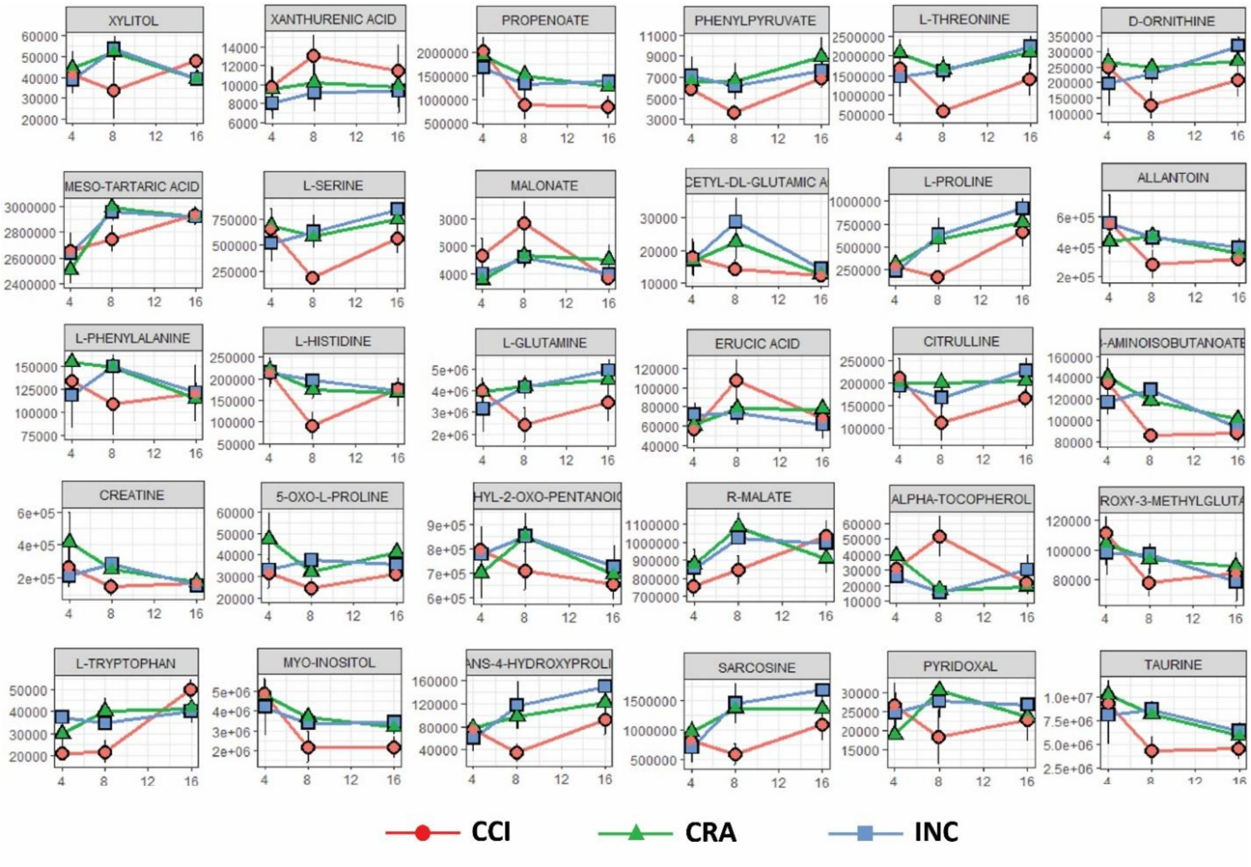

Figure S2. Plasma metabolite (RPIPLC-MS) profiles in the CCI mouse model of sTBI compared with sham controls (CRA, INC) across time points (4-16 h post-injury). The largest differences were detected at 8 h post-injury, where CCI mice showed clear divergence from controls, while 4 h and 16 h displayed smaller changes.

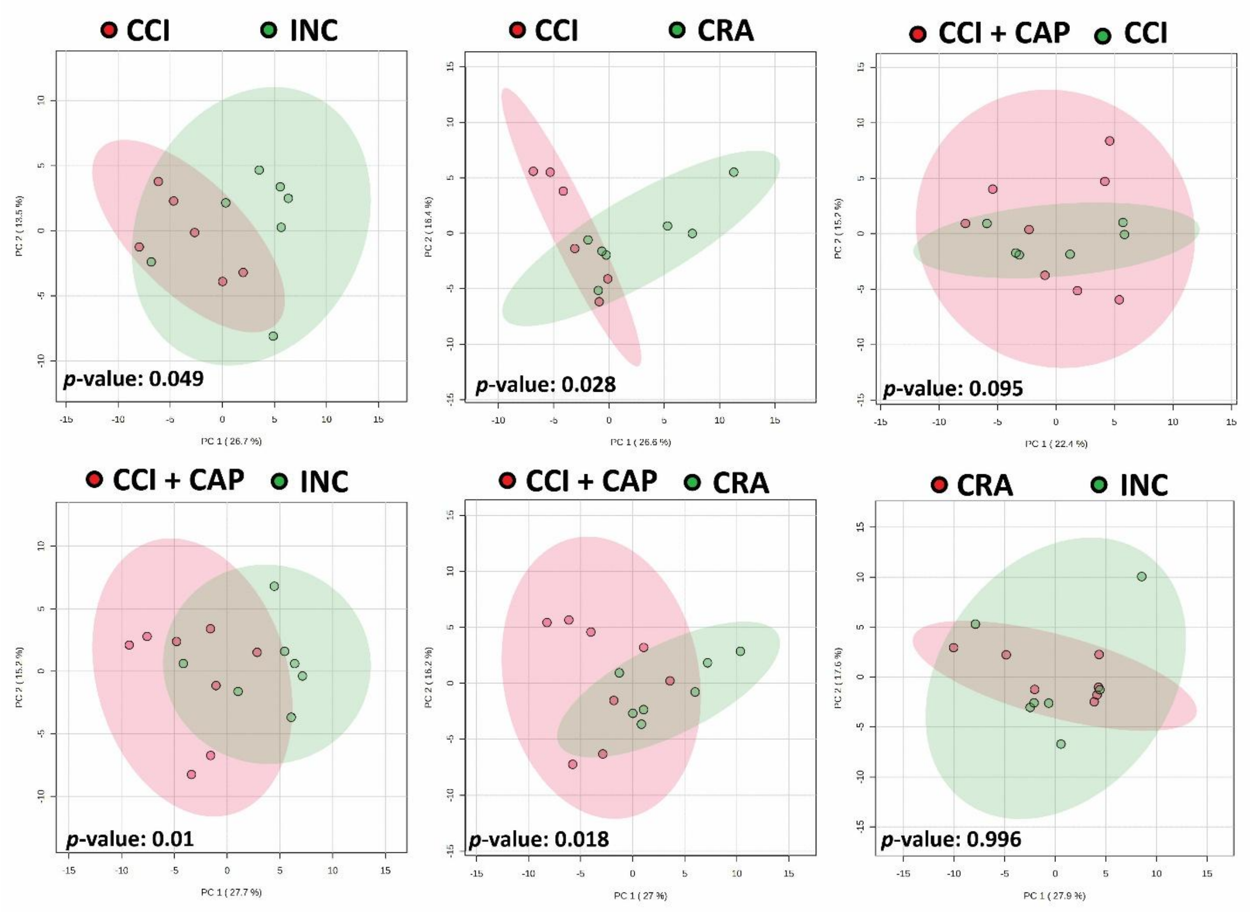

Figure S3. Principal component analysis (PCA) of plasma metabolic profiles in CCI, CCI-CAP, and sham control (INC and CRA) groups using HILIC data at 8h. The metabolic profiles of CCI and CCI-CAP mice differed significantly from those of sham controls, as shown by clear separations in the PCA space and supported by permutation test *p*-values (999 permutations). No significant differences were observed between the CCI and CCI-CAP groups, whereas CRA and INC exhibited comparable metabolic phenotypes, consistent with their sham status.

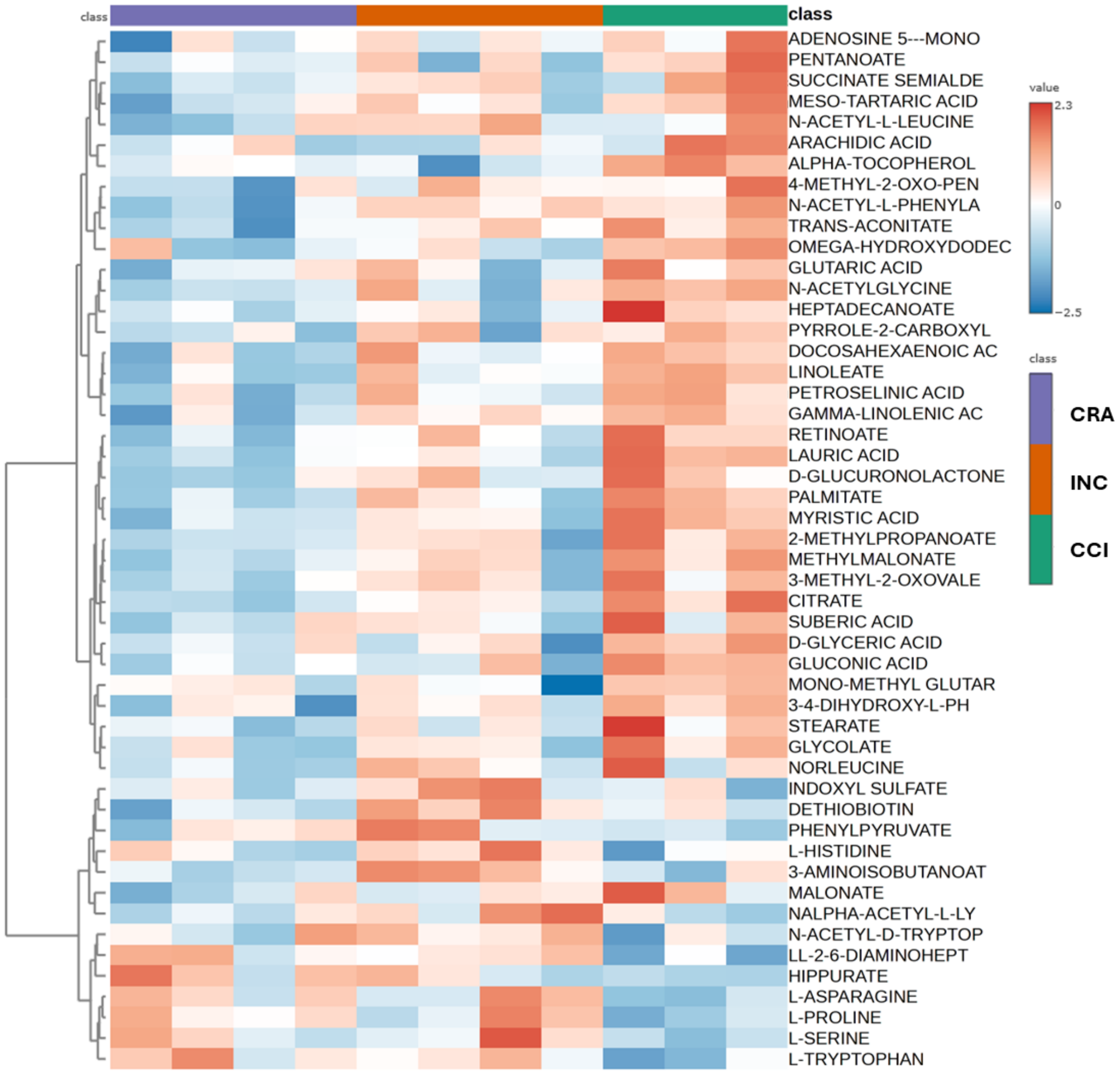

Figure S4. Heatmap of plasma metabolites identified by RPIPLC-MS in the CCI mouse model of sTBI compared with sham controls (CRA, INC). Distinct clustering patterns were observed at 8 h post-injury, where CCI mice exhibited the greatest divergence in metabolic profiles.

Name	Mean (SD) of CCI 8	Mean (SD) of INC 8	p-value	CCI 8/INC 8
FERULATE	-0.828 (0.844)	0.710 (0.363)	0.0011	Down
5-HYDROXYINDOLEACETATE	-0.898 (0.752)	0.770 (0.179)	0.0024	Down
ALPHA-HYDROXYISOBUTYRIC ACID	0.789 (0.435)	-0.676 (0.829)	0.0026	Up
3-METHYL-2-OXOVALERIC ACID	0.788 (0.525)	-0.675 (0.786)	0.0026	Up
SUCCINATE SEMIALDEHYDE	0.753 (0.383)	-0.646 (0.908)	0.005	Up
N-ACETYLGLYCINE	0.721 (0.525)	-0.618 (0.897)	0.0083	Up
D-RIBOSE	0.720 (0.449)	-0.617 (0.933)	0.0086	Up
OMEGA-HYDROXYDODECANOIC ACID	0.711 (0.672)	-0.609 (0.828)	0.0098	Up
L-PHENYLALANINE	0.694 (0.610)	-0.594 (0.893)	0.0125	Up
L-METHIONINE	-0.668 (0.430)	0.573 (1.008)	0.0176	Down
CORTISOL	-0.652 (0.905)	0.559 (0.726)	0.0214	Down
4-METHYL-2-OXO-PENTANOIC ACID	0.647 (0.701)	-0.554 (0.902)	0.0229	Up
N-ACETYL-L-ALANINE	0.635 (0.562)	-0.545 (0.994)	0.0261	Up
10-HYDROXYDECANOATE	0.630 (0.663)	-0.540 (0.947)	0.0279	Up
L-HISTIDINE	0.624 (0.412)	-0.535 (1.066)	0.0298	Up
XYLITOL	0.615 (0.814)	-0.527 (0.863)	0.0328	Up
N-ACETYL-L-PHENYLALANINE	-0.592 (1.058)	0.507 (0.646)	0.0418	Down
D-PANTOTHENIC ACID	-0.589 (0.808)	0.505 (0.901)	0.0431	Down
ALLANTOIN	0.381 (0.577)	-0.327 (1.205)	0.0350 (W)	Up

Table S1. T-test analysis of quantified plasma metabolites in **CCI vs. INC** at 8 h post-injury, HILIC-MS data.

Name	Mean (SD) of CCI 8	Mean (SD) of CRA 8	p-value	CCI 8/CRA 8
BENZYL ALCOHOL	0.841 (0.589)	-0.721 (0.630)	0.0008	Up
SUCCINATE SEMIALDEHYDE	0.831 (0.399)	-0.712 (0.765)	0.001	Up
L-METHIONINE	-0.800 (0.481)	0.686 (0.786)	0.002	Down
N-ACETYL-L-ALANINE	0.799 (0.854)	-0.685 (0.454)	0.0021	Up
ALPHA-HYDROXYISOBUTYRIC ACID	0.763 (0.421)	-0.654 (0.879)	0.0042	Up
3-METHYL-2-OXOVALERIC ACID	0.733 (0.564)	-0.629 (0.858)	0.0069	Up
CORTISOL	-0.703 (0.949)	0.603 (0.574)	0.0109	Down
OMEGA-HYDROXYDODECANOIC ACID	0.695 (0.724)	-0.596 (0.816)	0.0123	Up
D-RIBOSE	0.689 (0.500)	-0.590 (0.954)	0.0134	Up
N-ACETYLGLYCINE	0.680 (0.511)	-0.583 (0.961)	0.015	Up
ALLANTOIN	0.670 (0.931)	-0.574 (0.666)	0.0172	Up
HIPPURATE	-0.653 (1.068)	0.560 (0.507)	0.0211	Down
L-THREONINE	-0.635 (0.475)	0.544 (1.031)	0.0263	Down
MYO-INOSITOL	-0.630 (0.928)	0.540 (0.738)	0.0277	Down
10-HYDROXYDECANOATE	0.628 (0.439)	-0.538 (1.053)	0.0286	Up
XYLITOL	0.625 (0.948)	-0.536 (0.724)	0.0293	Up
D-PANTOTHENIC ACID	-0.586 (0.960)	0.502 (0.770)	0.0443	Down
L-PHENYLALANINE	0.579 (0.772)	-0.497 (0.938)	0.0474	Up
DL-5-HYDROXYLYSINE	-0.659 (0.747)	0.565 (0.853)	0.0082 (W)	Down

Table S2. T-test analysis of quantified plasma metabolites in **CCI vs. CRA** at 8 h post-injury, HILIC-MS data

Name	Mean (SD) of CAP 8	Mean (SD) of INC 8	p-value	CAP 8/INC 8
5-HYDROXYINDOLEACETATE	-0.761 (0.735)	0.869 (0.226)	0.0003	Down
ALPHA-HYDROXYISOBUTYRIC ACID	0.658 (0.625)	-0.752 (0.800)	0.0021	Up
N-ACETYLGLYCINE	0.651 (0.560)	-0.744 (0.870)	0.0024	Up
D-RIBOSE	0.636 (0.513)	-0.727 (0.932)	0.0034	Up
OMEGA-HYDROXYDODECANOIC ACID	0.591 (0.713)	-0.675 (0.862)	0.0082	Up
SUCCINATE SEMIALDEHYDE	0.644 (0.421)	-0.737 (0.970)	0.0083	Up
DEOXYCYTIDINE	0.587 (0.787)	-0.671 (0.792)	0.0088	Up
MEVALOLACTONE	0.586 (0.917)	-0.670 (0.607)	0.0089	Up
GLUTATHIONE	0.564 (0.769)	-0.644 (0.857)	0.0129	Up
CORTISOL	-0.560 (0.837)	0.640 (0.786)	0.0137	Down
N-ACETYL-L-ALANINE	0.556 (0.758)	-0.635 (0.884)	0.0147	Up
L-METHIONINE	-0.515 (0.610)	0.589 (1.069)	0.0266	Down
SN-GLYCEROL 3-PHOSPHATE	0.509 (0.679)	-0.581 (1.027)	0.0289	Up
L-PHENYLALANINE	0.491 (0.725)	-0.561 (1.015)	0.0362	Up
3-METHYL-2-OXOVALERIC ACID	0.707 (0.579)	-0.808 (0.718)	0.0012 (W)	Up
2-OXOADIPATE	0.507 (1.036)	-0.579 (0.588)	0.0140 (W)	Up
CREATINE	0.483 (0.880)	-0.552 (0.874)	0.0205 (W)	Up
4-METHYL-2-OXO-PENTANOIC ACID	0.396 (1.045)	-0.452 (0.782)	0.0289 (W)	Up
SARCOSINE	0.457 (0.844)	-0.522 (0.952)	0.0289 (W)	Up
FORMYL-L-METHIONYL PEPTIDE	0.539 (0.875)	-0.616 (0.781)	0.0289 (W)	Up

Table S3. T-test analysis of quantified plasma metabolites in **CCI +CAP vs. INC** at 8 h post-injury, HILIC-MS data

Name	Mean (SD) of CAP 8	Mean (SD) of CRA 8	p-value	CAP 8/CRA 8
SUCCINATE SEMIALDEHYDE	0.712 (0.437)	-0.813 (0.814)	0.0005	Up
BENZYL ALCOHOL	0.666 (0.807)	-0.761 (0.554)	0.0017	Up
ALPHA-HYDROXYISOBUTYRIC ACID	0.640 (0.609)	-0.732 (0.854)	0.0031	Up
L-METHIONINE	-0.622 (0.680)	0.711 (0.829)	0.0045	Down
N-ACETYLGLYCINE	0.620 (0.550)	-0.709 (0.939)	0.0047	Up
D-RIBOSE	0.608 (0.568)	-0.695 (0.949)	0.006	Up
N-ACETYL-L-ALANINE	0.614 (0.988)	-0.701 (0.346)	0.0066	Up
CORTISOL	-0.600 (0.903)	0.686 (0.594)	0.0069	Down
SN-GLYCEROL 3-PHOSPHATE	0.595 (0.700)	-0.680 (0.866)	0.0076	Up
OMEGA-HYDROXYDODECANOIC ACID	0.571 (0.766)	-0.652 (0.848)	0.0116	Up
DL-5-HYDROXYLYSINE	-0.531 (0.778)	0.607 (0.907)	0.0213	Down
L-ASPARAGINE	-0.503 (0.703)	0.575 (1.017)	0.0313	Down
THYMIDINE	0.477 (0.980)	-0.545 (0.752)	0.0434	Up
RESORCINOL MONOACETATE	0.467 (0.944)	-0.534 (0.818)	0.0485	Up
3-METHYL-2-OXOVALERIC ACID	0.668 (0.619)	-0.764 (0.781)	0.0037 (W)	Up
FORMYL-L-METHIONYL PEPTIDE	0.465 (0.928)	-0.531 (0.843)	0.0401 (W)	Up

Table S4. T-test analysis of quantified plasma metabolites in **CCI + CAP vs. CRA** at 8 h post-injury, HILIC-MS data

**Name**	**Mean (SD) of CCI 7**	**Mean (SD) of INC 7**	**p-value**	**CCI 7/INC 7**
L-METHIONINE	-1.017 (0.988)	0.508 (0.516)	0.0049	Down
XYLITOL	0.936 (0.876)	-0.468 (0.701)	0.0127	Up
SN-GLYCEROL 3-PHOSPHATE	0.752 (0.158)	-0.376 (1.038)	0.0179	Up
DEOXYCYTIDINE	0.844 (0.608)	-0.422 (0.895)	0.0303	Up
L-THREONINE	-0.796 (0.745)	0.398 (0.889)	0.0445	Down
TAURINE	0.792 (0.878)	-0.396 (0.839)	0.0457	Up
L-ANSERINE	0.625 (0.226)	-0.312 (1.102)	0.0493	Up

Table S5. T-test analysis of plasma metabolites in **CCI vs. INC** at 7 days post-injury, HILIC-MS data

**Name**	**Mean (SD) of CCI 7**	**Mean (SD) of CRA 7**	**p-value**	**CCI 7/CRA 7**
SN-GLYCEROL 3-PHOSPHATE	0.846 (0.243)	-0.483 (0.942)	0.009	Up
TAURINE	0.861 (0.881)	-0.492 (0.709)	0.0207	Up
L-TRYPTOPHAN	-0.818 (0.794)	0.467 (0.807)	0.031	Down
L-TYROSINE	-0.774 (1.059)	0.442 (0.691)	0.0445	Down

Table S6. T-test analysis of plasma metabolites in **CCI vs. CRA** at 7 days post-injury, HILIC-MS data

**Name**	**Mean (SD) of CAP 7**	**Mean (SD) of INC 7**	**p-value**	**CAP 7/INC 7**
SUCCINATE	-0.561 (0.591)	0.561 (1.036)	0.0185	Down
DEOXYURIDINE	0.522 (0.995)	-0.522 (0.727)	0.0311	Up

Table S7. T-test analysis of plasma metabolites in **CCI +CAP vs. INC** at 7 days post-injury, HILIC-MS data

**Name**	**Mean (SD) of CAP 7**	**Mean (SD) of CRA 7**	**p-value**	**CAP 7/CRA 7**
SUCCINATE	-0.577 (0.761)	0.742 (0.765)	0.004	Down
L-TRYPTOPHAN	-0.519 (0.920)	0.667 (0.668)	0.0125	Down
N-METHYL-D-ASPARTIC ACID	-0.516 (0.848)	0.663 (0.793)	0.0132	Down
N-ACETYL-L-PHENYLALANINE	-0.462 (1.100)	0.593 (0.397)	0.0228	Down
TAURINE	0.475 (0.837)	-0.610 (0.891)	0.0254	Up
N-ALPHA-ACETYL-L-ASPARAGINE	-0.441 (1.092)	0.567 (0.493)	0.0406	Down
SUCCINATE SEMIALDEHYDE	0.414 (1.153)	-0.532 (0.373)	0.0434	Up
FERULATE	-0.430 (1.018)	0.553 (0.696)	0.0468	Down

Table S8. T-test analysis of plasma metabolites in **CCI + CAP vs. CRA** at 7 days post-injury, HILIC-MS data.

Name	Mean (SD) of CRA 8 hours	Mean (SD) of CCI 8 hours	p-value	q-value (FDR)	CRA/TBI
N-ACETYLGLYCINE	-0.782 (0.261)	1.043 (0.206)	0.0002	0.0232	Down
ALPHA-TOCOPHEROL	-0.758 (0.306)	1.011 (0.420)	0.0013	0.0797	Down
PALMITATE	-0.744 (0.374)	0.991 (0.459)	0.0026	0.0797	Down
LAURIC ACID	-0.733 (0.448)	0.978 (0.434)	0.0039	0.0797	Down
GLUCONIC ACID	-0.731 (0.501)	0.975 (0.359)	0.0042	0.0797	Down
MYRISTIC ACID	-0.729 (0.441)	0.973 (0.475)	0.0045	0.0797	Down
CITRATE	-0.729 (0.274)	0.973 (0.635)	0.0045	0.0797	Down
LINOLEATE	-0.726 (0.577)	0.969 (0.195)	0.0049	0.0797	Down
METHYLMALONATE	-0.722 (0.330)	0.963 (0.634)	0.0056	0.0806	Down
L-PROLINE	0.687 (0.550)	-0.916 (0.585)	0.0137	0.1763	Up
D-GLYCERIC ACID	-0.665 (0.676)	0.887 (0.497)	0.0209	0.1992	Down
DOCOSAHEXAENOIC ACID	-0.663 (0.761)	0.885 (0.276)	0.0216	0.1992	Down
TRANS-ACONITATE	-0.658 (0.681)	0.877 (0.534)	0.0237	0.1992	Down
PYRROLE-2-CARBOXYLATE	-0.653 (0.711)	0.870 (0.503)	0.0258	0.1992	Down
MESO-TARTARIC ACID	-0.653 (0.665)	0.870 (0.591)	0.0258	0.1992	Down
N-ACETYL-L-PHENYLALANINE	-0.653 (0.689)	0.870 (0.548)	0.0259	0.1992	Down
PETROSELINIC ACID	-0.652 (0.740)	0.869 (0.443)	0.0262	0.1992	Down
GAMMA-LINOLENIC ACID	-0.641 (0.812)	0.855 (0.304)	0.0309	0.2217	Down
L-ASPARAGINE	0.637 (0.769)	-0.849 (0.470)	0.033	0.2239	Up
HEPTADECANOATE	-0.630 (0.387)	0.840 (0.960)	0.0361	0.2328	Down
GLYCOLATE	-0.627 (0.681)	0.836 (0.687)	0.0379	0.2331	Down
3-METHYL-2-OXOVALERIC ACID	-0.622 (0.482)	0.829 (0.921)	0.0405	0.2372	Down
3-4-DIHYDROXYBENZOATE	0.611 (0.619)	-0.815 (0.826)	0.0464	0.26	Up

Table S9. T-test analysis of plasma metabolites in **CCI vs. CRA** at 8 hours post-injury, RPIPLC-MS data

Name	Mean (SD) of INC 8 hours	Mean (SD) of CCI 8 hours	p-value	q-value (FDR)	INC/TBI
LAURIC ACID	-0.686 (0.583)	0.915 (0.542)	0.014	0.5752	Down
OMEGA-HYDROXYDODECANOIC ACID	-0.669 (0.715)	0.891 (0.383)	0.0197	0.5752	Down
URIDINE	-0.652 (0.819)	0.870 (0.089)	0.0329	0.5752	Down
CITRATE	-0.622 (0.583)	0.829 (0.828)	0.0404	0.5752	Down
L-TRYPTOPHAN	0.610 (0.530)	-0.813 (0.917)	0.047	0.5752	Up
DETHIOBIOTIN	0.608 (0.755)	-0.810 (0.650)	0.0485	0.5752	Up

Table S10. T-test analysis of plasma metabolites in **CCI vs. INC** at 8 hours post-injury, RPIPLC-MS data.

References

1. N. Osier, C. E. Dixon, The Controlled Cortical Impact Model of Experimental Brain Trauma: Overview, Research Applications, and Protocol. *Methods Mol Biol* **1462**, 177-192 (2016).
2. J. Romine, X. Gao, J. Chen, Controlled cortical impact model for traumatic brain injury. *J Vis Exp*, e51781-e51781 (2014).
3. M. M. Banoei *et al.*, Metabolomic and metallomic profile differences between Veterans and Civilians with Pulmonary Sarcoidosis. *Scientific Reports* **9**, 19584 (2019).
4. M. F. Clasquin, E. Melamud, J. D. Rabinowitz, LC-MS Data Processing with MAVEN: A Metabolomic Analysis and Visualization Engine. *Current protocols in bioinformatics / editoral board, Andreas D. Baxevanis ... [et al.]* **0 14**, Unit14.11-Unit14.11 (2012).
5. E. Melamud, L. Vastag, J. D. Rabinowitz, Metabolomic analysis and visualization engine for LC-MS data. *Analytical chemistry* **82**, 9818-9826 (2010).
6. S. Agrawal *et al.*, El-MAVEN: A Fast, Robust, and User-Friendly Mass Spectrometry Data Processing Engine for Metabolomics. *Methods Mol Biol* **1978**, 301-321 (2019).
7. J. Chong, D. S. Wishart, J. Xia, Using MetaboAnalyst 4.0 for Comprehensive and Integrative Metabolomics Data Analysis. *Current Protocols in Bioinformatics* **68**, e86 (2019).

Members of CCCTBG

**No.**	**Member**	**Affiliation / location listed or identifiable from CCCTG/CCCTBG profile**	**City**
1	Patricia Liaw	McMaster University	Hamilton
2	Jamie S. Hutchison	University of Toronto / The Hospital for Sick Children	Toronto
3	Alison Fox-Robichaud	McMaster University	Hamilton
4	Sean Gill	Western University / University of Western Ontario	London
5	Asher Mendelson	University of Manitoba / Health Sciences Centre	Winnipeg
6	Taylor Kain	University of Toronto	Toronto
7	Jane Batt	University of Toronto / St. Michael's Hospital	Toronto
8	Brent Winston	University of Calgary	Calgary
9	Anne-Marie Guerguerian	The Hospital for Sick Children / University of Toronto	Toronto
10	Gloria Vazquez Grande	University of Manitoba Health Sciences Centre / St. Boniface	Winnipeg
11	Jan-Alexis Hôpital Tremblay	Université de Montréal / Hôpital Maisonneuve-Rosemont	Montréal
12	John Boyd	St. Paul's Hospital / University of British Columbia	Vancouver
13	John Marshall	University of Toronto / Unity Health Toronto	Toronto
14	John Muscedere	Kingston Health Sciences Centre / Queen's University	Kingston
15	Lauralyn McIntyre	The Ottawa Hospital / University of Ottawa / Ottawa Hospital Research Institute	Ottawa

16	Lorenzo Del Sorbo	Toronto General Hospital / University of Toronto	Toronto
17	Sheldon Magder	Royal Victoria Hospital / McGill University	Montréal

Members of CTRC

No.	Member	Affiliation/location listed on CTRC profile	City
1	Amanda Black	University of Calgary	Calgary
2	Patrick Archambault	CISSS de Chaudière-Appalaches	Lévis
3	Miriam Beauchamp	Université de Montréal	Montréal
4	Carolina Bottari	Université de Montréal	Montréal
5	Matthew Burke	University of Toronto	Toronto
6	David Clarke	Dalhousie University	Halifax
7	Chantel Debert	Foothills Medical Centre	Calgary
8	Sean Dukelow	University of Calgary	Calgary
9	Carolyn Emery	University of Calgary	Calgary
10	Pierre Frémont	Université Laval	Québec
11	Isabelle Gagnon	McGill University Health Centre (MUHC)	Montréal
12	Mauricio Garcia-Barrera	University of Victoria	Victoria
13	Christopher Grant	University of Calgary	Calgary
14	Robin Green	KITE Research Institute, Toronto Rehab–University Health Network / University of Toronto	Toronto
15	Dinesh Kumbhare	University of Toronto	Toronto
16	Mark MacLean	Dalhousie University	Halifax
17	Tatyana Mollayeva	KITE Research Institute, University Health Network / University of Toronto	Toronto
18	William Panenka	University of British Columbia – Vancouver campus	Vancouver
19	Mark Paramlall	Dalhousie University	Halifax
20	Vickie Plourde	University of Moncton	Moncton
21	Marie-Julie Potvin	UQAM	Montréal
22	Scott Ramsay	University of British Columbia – Vancouver campus	Vancouver
23	Nick Reed	University of Toronto	Toronto
24	Kathryn Schneider	University of Calgary	Calgary
25	Sandy Shultz	Vancouver Island University	Nanaimo

26	Jon Smirl	University of Calgary	Calgary
27	Chand Taneja	Island Health – Queen Alexandra Centre for Children's Health	Victoria
28	Allen Thornton	Simon Fraser University	Burnaby
29	Alexis Turgeon	CHU de Québec – Université Laval, Hôpital de l'Enfant-Jésus	Québec
30	Anne Wheeler	The Hospital for Sick Children	Toronto
31	Brent Winston	University of Calgary	Calgary
32	Keith Yeates	University of Calgary	Calgary
33	Roger Zemek	Children's Hospital of Eastern Ontario (CHEO)	Ottawa
